# Supplementary material for: Therapeutic Potential of Natural Compounds in Neurodegenerative Diseases: Insights from Clinical Trials
Source: Pharmaceutics. 2023 Jan 7;15(1):212. doi: 10.3390/pharmaceutics15010212 (PMC9860553; doi:10.3390/pharmaceutics15010212)
Supplement: Supplementary file 1 [file pharmaceutics-15-00212-s001.zip › pharmaceutics-2102056-supplementary.pdf]

pharmaceutics

Supplementary Materials: Therapeutic Potential of Natural Compounds in Neurodegenerative Diseases: Insights from Clinical Trials

Stéphanie Andrade, Débora Nunes, Meghna Dabur, Maria J. Ramalho, Maria C. Pereira and Joana A. Loureiro

List of natural compounds included in the literature search:.

1,2,3,4,6-Penta-O-galloyl-β-d-glucopyranose
13-Desmethyl spirolide C
β-carotene
Apigenin
Asiatic acid
Baicalein
Berberine
Bryostatin
Docosahexaenoic acid
Caffeic acid
Caffeine
Crocin
Curcumin
Ellagic acid
Enoxaparin
Epicatechin
Epigallocatechin gallate
Ferulic acid
Fisetin
Gallic acid
Glycine betaine
Gossypin
Gypenoside
Homotaurine
Honokiol
Huperzine A
Hydroxytyrosol
Kaempferol
Lipoic acid
l-theanine
Luteolin
Melatonin
Morin
Myricetin
Naringenin
Naringin
Nicotine
Nordihydroguaiaretic acid
Oleocanthal
Oleuropein
Osthole
Piperine
Puerarin

Punicalagin
Quercetin
Resveratrol
Retinoic acid
Rhodosin
Rosmarinic acid
Rutin
Salvianolic acid B Tannic acid
Scyllo-inositol
Vanillic acid
Viniferin
Vitamin A
Vitamin B
Vitamin C
Vitamin D
Vitamin E
Vitamin K
